# Evaluation of Metronidazole Resistance of Vaginal Swab Isolates from South African Women Treated for Bacterial Vaginosis

**DOI:** 10.3390/antibiotics13121217

**Published:** 2024-12-14

**Authors:** Timo Schwebs, Ann-Katrin Kieninger, Lenka Podpera Tisakova, Vera Oberbauer, Rocío Berdaguer, Andile Mtshali, Gugulethu Mzobe, Anne Rompalo, Adrian Mindel, Marothi Letsoalo, Nigel Garrett, Sinaye Ngcapu, Lorenzo Corsini

**Affiliations:** 1 BioNTech R&D (Austria) GmbH, Helmut-Qualtinger-Gasse 2, 1030 Vienna, Austria; 2 Centre for the AIDS Programme of Research in South Africa (CAPRISA), Durban 4013, South Africa; 3 Department of Medical Microbiology, University of KwaZulu-Natal, Durban 4013, South Africa; 4 Department of Gynecology and Obstetrics, Johns Hopkins University, Baltimore, MD 21287, USA; 5 Discipline of Public Health Medicine, University of KwaZulu-Natal, Durban 4001, South Africa

**Keywords:** bacterial vaginosis, vaginal microbiome, community state types, *Gardnerella*, *Lactobacillus*, metronidazole, antibiotic resistance, MIC, vaginal swabs

## Abstract

**Background/Objectives**: The high recurrence rate of bacterial vaginosis (BV) after antibiotic treatment is at least partially attributed to resistant bacteria. The CAPRISA 083 (CAP083) study investigated the influence of metronidazole (MTZ) treatment on the vaginal microbiome in 56 South African women diagnosed with BV. To explore the etiology of recurrent BV in this cohort, we retrospectively analyzed vaginal swabs collected in CAP083 before and after MTZ treatment. **Methods**: We isolated over 1200 bacterial strains, including *Gardnerella, Lactobacillus*, *Prevotella,* and *Fannyhessa*, and determined the minimum inhibitory concentration (MIC) of MTZ and the resistance status according to CLSI and EUCAST guidelines. **Results**: At baseline, 64% (CLSI) of *Gardnerella* isolates were resistant to MTZ, rising to 80% after MTZ treatment by the 12-week visit. *Lactobacillus* species consistently exhibited resistance of 100%, while *Fannyhessea vaginae* maintained resistance rates of 78–91% across visits. *Prevotella* strains varied, showing two susceptible isolates at baseline and one resistant isolate at the 6-week visit. Susceptible and resistant *Gardnerella* isolates were often isolated from the same swab, and 70% (CLSI) of participants had at least one resistant *Gardnerella* strain already at baseline. Sensitive *Gardnerella* isolates were not a predictor of an MTZ-mediated reduction in *Gardnerella* abundance. **Conclusions**: Our data indicate that the 23% cure rate in CAP083 was associated with a combination of a high share of MTZ-resistant bacteria at baseline, a potentially insufficient MTZ dose regimen, and a constantly high average abundance of *Gardnerella*. Future research should explore novel therapeutic strategies to enhance treatment efficacy and combat antibiotic resistance.

## 1. Introduction

Bacterial vaginosis (BV) is the most prevalent vaginal condition among women of reproductive age, affecting approximately 30% of this population globally [1,2]. BV is particularly prevalent among women of childbearing age in sub-Saharan Africa, with rates of 42.1% in those aged 15–24 and 41.2% in those aged 25–49 [3]. Clinically, BV manifests through symptoms such as increased vaginal discharge with a distinctive fish-like odor, vaginal discomfort, and/or urinary symptoms. BV is characterized by a reduction in beneficial *Lactobacillus* species and by colonization with a diverse spectrum of primarily anaerobic bacteria [4]. BV is associated with a range of negative sexual and reproductive outcomes, such as a higher risk of preterm birth, miscarriage, increased genital inflammation, sexually transmitted infections (STIs), and higher susceptibility to HIV [5,6]. Treatment failure and relapse in up to 60% of patients within 6–12 months [7,8], leading to recurrent (≥3 BV episodes per year) or refractory (no response upon antibiotic treatment) BV, creates a high burden of disease and impairs the quality of life of affected women [9].

Vaginal microbiome communities are complex and can be classified into five community state types (CSTs) [10], although intermediate states and transitions within the menstrual cycle have been described [11,12]. CSTs are characterized by the abundance of specific *Lactobacillus* species, except for CST IV, which has high diversity [10]. CST IV is commonly associated with BV, but also the *L. iners*-abundant CST III is considered imbalanced and often shifts into the BV state [13,14,15]. The predominance of non-*iners* lactobacilli is less commonly observed among African women compared to Caucasian women, highlighting geographical and ethnic differences in vaginal microbiome composition [16,17]. During the development of BV, the vaginal microbiome shifts towards increased bacterial diversity, particularly of anaerobic microbes such as *Gardnerella*, *Prevotella*, *Fannyhessea* (formerly *Atopobium*), *Mobiluncus*, and BV-associated bacteria 1 (BVAB1) (Candidatus *Lachnocurva vaginae* [18]) and BVAB2 and 3 [19]. The formation of a *Gardnerella*-dominated biofilm was described as characteristic of BV [5] and provides favorable conditions for other anaerobic bacteria to proliferate [19,20]. A symbiotic relationship between *Gardnerella* and *Prevotella* strains further establishes the biofilm and degrades the vaginal mucous layer, which in turn increases the adherence of other BVAB [19].

Although it is not always classified as an infection, clinically apparent BV is usually treated with oral or topical metronidazole (MTZ) or clindamycin (CLI) as the standard of care, with emerging experimental alternatives including antiseptic treatments, *Lactobacillus*-based live biotherapeutics, antimicrobial agents such as endolysins, pro- and prebiotics, and vaginal microbiome transplantation [21]. Several studies have shown a modest reduction in the bacterial abundances of BV-associated microorganisms after antibiotic treatment. While this was inversely associated with increased relative abundances of *Lactobacillus* species [13,22,23], often this increase is largely represented by *L. iners*, which has been described as destabilizing itself [1,13,24,25]. The high rate of BV recurrence of 60% may be caused by the inability of antibiotics to eliminate the biofilm [20,26] but also by antibiotic resistance [2,27]. *In vitro* passaging experiments with sub-minimum inhibitory concentration (MIC) concentrations of MTZ showed that resistance developed after 5–10 passages in six out of six tested *Gardnerella* strains [28]. Previously, we reported that 12/20 (60%) *Gardnerella* strains isolated from women with BV were resistant to MTZ, whereas all were susceptible to CLI when grown in the planktonic form [29]. However, when grown as biofilm *in vitro*, CLI was ineffective against seven out of nine *Gardnerella* strains [28]. In a larger study by Petrina et al. (2017), which analyzed 605 BV-related bacterial strains, all 110 *Gardnerella* isolates demonstrated sensitivity to CLI, indicating its potential effectiveness in treating infections caused by this genus [30]. However, 27% of the isolates exhibited resistance to MTZ. Furthermore, this study reported that all 25 *Fannyhessea* isolates were also sensitive to CLI, yet 18% showed resistance to MTZ [31], suggesting that MTZ may not be a reliable treatment option for a significant portion of these bacteria.

Previous research by Mtshali et al. (2021) demonstrated that treatment with MTZ was associated with a gradual shift of vaginal microbiome profiles away from predominance of strict anaerobes towards an increased abundance of *L. iners* in the CAPRISA 083 (CAP083) clinical trial [13]. Participants were diagnosed for BV by both Nugent Score (NS) and assessment of clinical symptoms, and were treated according to the South African Management Guidelines for sexually transmitted infections [32]. All enrolled 56 participants completed two follow-up visits at 6 and 12 weeks. The limited efficacy of MTZ in fully eradicating BV-related pathogens raises important concerns regarding treatment success and the potential for recurrence. This highlights the necessity of examining the bacterial resistance profile in the vaginal microbiome of the CAP083 participants, as this may influence treatment outcomes and the persistence of BV.

In the CAP083 study [13,33], intermediate BV was defined as NS = 4–6, BV as NS ≥ 7, and NS ≤ 3 was considered BV negative. At the baseline visit, all participants received a single oral dose of 2 g MTZ. At the 6-week visit, participants with a NS ≥ 4 (39/56) received further treatment with 400 mg MTZ orally daily for 5 days. At the second follow-up visit, 12 weeks after treatment, a microbiological cure rate (NS ≤ 3) of 23% was reported. At all three visits, vaginal swabs were taken prior to treatment to investigate genital inflammation markers and changes in the vaginal microbiota in response to MTZ treatment by 16S rRNA sequencing.

Pharmacokinetic (PK) studies of MTZ conducted by other groups reported a maximum plasma concentration (c_max_) of ~40 µg/mL after oral application of 2 g MTZ, dropping to 4–5 µg/mL after 24 h [31,34]. In another study, oral application of 400 mg MTZ twice daily for 7 days resulted in a steady-state concentration of 6.9 µg/mL in plasma [35]. We used these values to interpret MIC values measured for bacterial isolates from CAP083.

In the present study, we isolated bacterial strains from vaginal swabs from CAP083, that is from women with laboratory-diagnosed STIs and/or BV (NS ≥ 4), both prior to and following MTZ exposure. We characterized the resistance levels of the vaginal microbiota and compared the prevalence of MTZ-resistant strains in women in whom treatment failed (NS ≥ 4) with those who were microbiologically cured (NS ≤ 3). Through this investigation, our research sought to enhance understanding of MTZ resistance patterns and their implications for treatment effectiveness, ultimately contributing to the development of improved strategies for managing BV.

## 2. Results

### 2.1. More than 1200 Bacterial Strains from over 80 Species Were Isolated from 168 Vaginal Swabs of 56 CAP083 Study Participants

**Figure 1:**
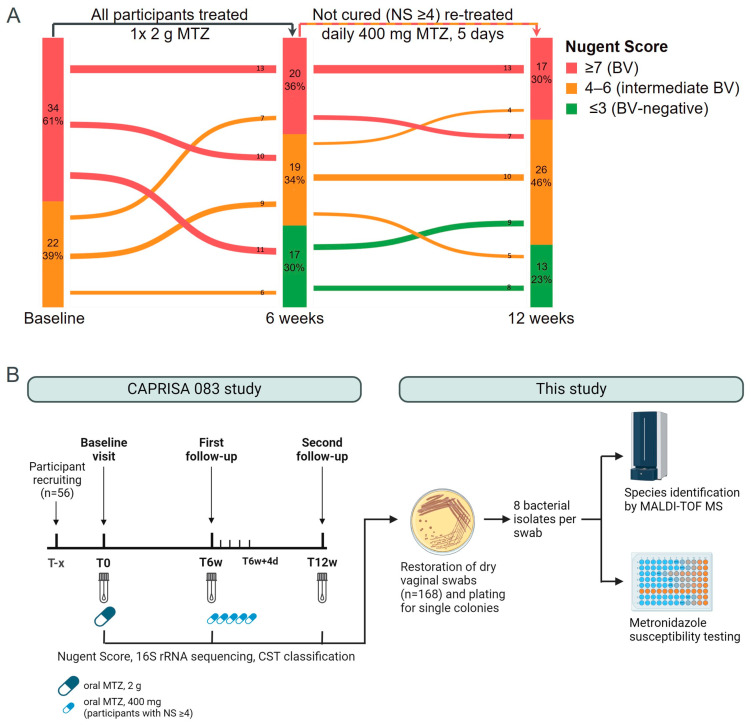
Transition of BV states defined by NS across visits and study overview. (**A**) Alluvial diagram showing the NS of participants at each visit and the transitioning of participants between NS groups. Data are from [13]. The number and percentage of participants in each group are stated within the bars. (**B**) The vaginal swabs analyzed in this study were collected in the CAP083 study [13]. At the baseline visit the participants received 2 g MTZ orally. At the 6-week visit (T6w), participants with a NS ≥ 4 were treated with daily 400 mg MTZ orally for 5 days, and after 12 weeks (T12w), a second follow-up visit was conducted. At each visit, the NS was determined, and vaginal swabs were collected for 16S rRNA sequencing and CST classification. Dry vaginal swabs stored at −80 °C were then used in this study to isolate bacterial strains. From each swab, eight bacterial colonies were isolated and identified by MALDI-TOF MS. All isolates were tested for MTZ susceptibility in a microbroth dilution assay. CST—community state type; NS—Nugent Score; MALDI-TOF MS—matrix-assisted laser desorption/ionization mass spectrometry; MTZ—metronidazole.

First, we re-analyzed the transition of study participants between BV statuses defined by NS across the three visits of CAP083 (Figure 1A). At enrollment of CAP083, 34 (61%) participants had NS ≥ 7 (BV positive), and 22 (39%) had NS = 4–6 (intermediate BV). All participants received a single 2 g oral dose of MTZ, but only 17 (30%) participants had NS ≤ 3 (BV negative) at the 6-week visit (initially cured). After the 6-week visit, the 20 (36%) participants who still had NS ≥ 7 and the 19 (34%) who had a NS of 4–6 were treated with daily doses of 400 mg oral MTZ for 5 days. Of these 39 participants, only 5 (9% of total) transitioned to the NS ≤ 3 group (cured), whereas 34 (61% of total) participants still had NS ≥ 4 at the 12-week visit (persistent BV). Of the 17 participants who were initially cured at the 6-week visit and therefore not retreated, 9 participants reverted to intermediate BV at the 12-week visit (recurrent BV). Overall, 13 (23%) of participants were BV negative at the 12-week visit, whereas 26 (46%) and 17 (30%) had NS = 4–6 and NS ≥ 7, respectively. After reconstituting leftover vaginal swabs from each participant at each visit of CAP083 [13] on blood agar, we randomly picked eight bacterial colonies per sample, identified them by MALDI-TOF MS, and tested their susceptibility to metronidazole (MTZ) (Figure 1B).

**Figure 2:**
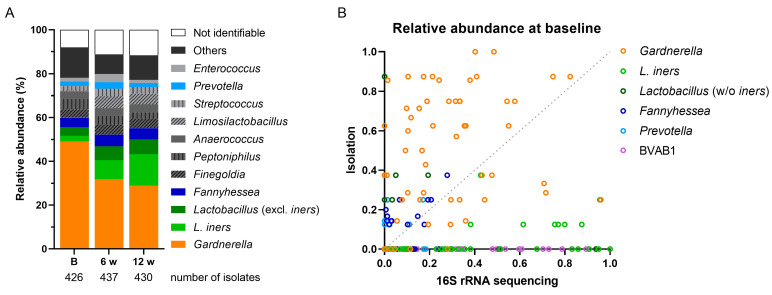
Relative abundance of top ten isolated bacterial genera and correlation to 16S rRNA sequencing. (**A**) Bacterial isolates from all participants relative to the total number of isolates per visit (stated below the bars). The genus *Lactobacillus* is segmented into *Lactobacillus* excluding *L. iners* (dark green) and *L. iners* only (light green). Genera belonging to the class Clostridia are shown in dark gray; genera of the order Lactobacillales (except *Lactobacillus*) are shown in light gray. Isolates that could not be identified are labeled as “not identifiable”. (**B**) Correlation of the relative abundance of the stated genera between the isolated bacteria and the bacteria identified by 16S rRNA sequencing of the vaginal swabs [13]. Isolates of *Prevotella bivia* and *P. timonensis* are correlated to sequence reads for *P. bivia*. Dotted line shows the line of identity. B—baseline.

Across all three visits, we isolated a total of 1293 bacterial strains covering 39 genera and 82 species. The relative abundance of the isolates by genus is shown in Figure 2A. Overall, the most abundant genus isolated was *Gardnerella*, comprising almost 50% (209/426) of bacteria at baseline and 34% (139/437) and 29% (124/430) at the 6-week and 12-week visits, respectively. *Lactobacillus* was the second most abundant genus among the isolates (185/1293), and there was an increasing relative abundance of *L. iners* from baseline (11/426) to follow-up visits (38/437 and 62/430 at 6 and 12 weeks, respectively), supporting the conclusion by Mtshali and colleagues that MTZ treatment enhances *L. iners* abundance [13]. Other BV-associated pathogens, including *Fannyhessea* and *Prevotella,* were also isolated (Figure 2A).

Next, we compared the relative abundances of bacterial genera determined by culturing isolates in this study to the results from 16S rRNA sequencing by Mtshali et al. (2021). The distribution of bacteria isolated in this study was consistent with the CSTs identified by 16S rRNA gene sequencing [13], with *L. iners* predominantly found in CST III, *Gardnerella* strains primarily isolated from CST IV, and lower lactobacilli counts among CST IV isolates, consistent with the CST definitions (Figure S1). We found that, compared with the 16S rRNA analysis, *Gardnerella* was overrepresented among cultured isolates, while *L. iners* was underrepresented, and BVAB1, frequently detected by sequencing, could not be cultured at all (Figure 2B).

### 2.2. Most Bacterial Isolates from Study Participants Were Resistant to MTZ

**Figure 3:**
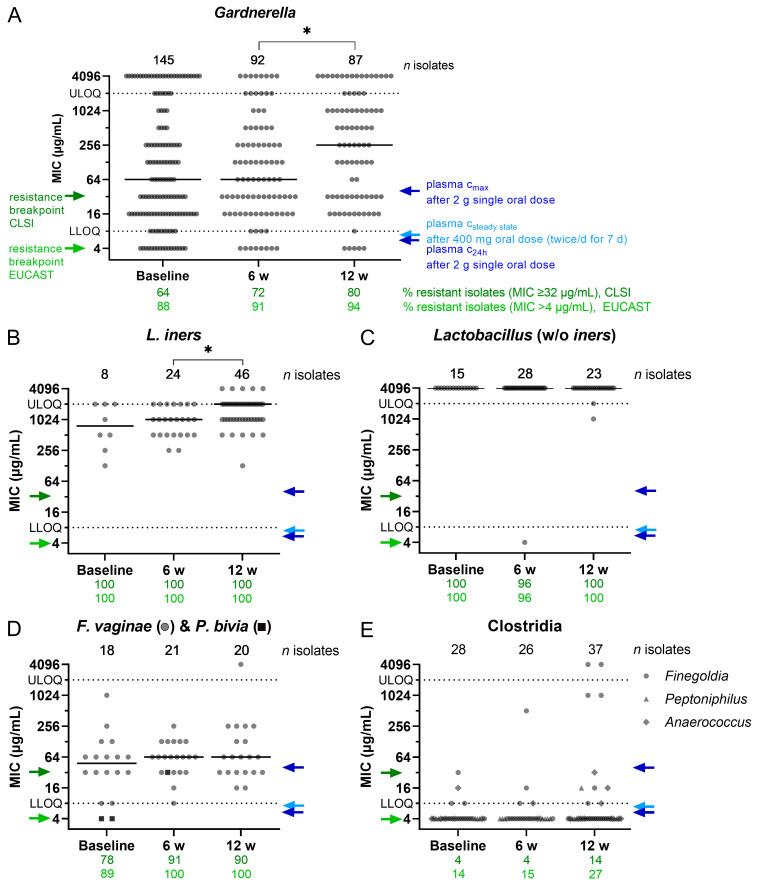
Minimum inhibitory concentration of metronidazole on the top ten isolated genera across visits. (**A**–**E**) The MICs of MTZ were determined for isolated strains from the visit stated at the x-axis. The number of tested isolates (*n*) is stated above the graphs. The median is shown in (**A**–**D**) as a solid line. Values above or below the LOQ (dotted line) were arbitrarily set to 4096 µg/mL or 4 µg/mL, respectively. The c_max_ and c_24h_ plasma concentrations (40 µg/mL and 4–7 µg/mL, respectively [31,34]) after a single oral dose of 2 g MTZ are indicated by dark blue arrows, and the plasma steady-state concentration c_steady_ state after a 400 mg oral dose (twice/d for 7 d; 6.9 µg/mL [35]) is indicated by a light blue arrow. MTZ resistance breakpoints based on CLSI (dark green arrow) and EUCAST (light green arrow) are indicated on the left. Percentages of resistant isolates per visit are stated below the graphs. An ordinary one-way ANOVA was performed on log2-transformed MIC values followed by Šidák’s multiple comparisons test to compare baseline to the 6-week visit and the 6-week visit to the 12-week visit. Statistically significant differences between at least two groups were revealed in (**A**) F(2,321) = 4.379, *p* = 0.0133; p(6 w vs. 12 w) = 0.0170 = *, and (**B**) F(2,75) = 4.954, *p* = 0.0095; p(6 w vs. 12 w) = 0.0315 = *. LLOQ—lower limit of quantification; MIC—minimum inhibitory concentration; ULOQ—upper limit of quantification; *L. iners*—*Lactobacillus iners*; *F. vaginae*—*Fannyhessea vaginae*; *P. bivia*—*Prevotella bivia*.

To evaluate whether bacterial resistance against MTZ emerges after treatment, all isolates were analyzed in a semi-automated microdilution broth assay. The MIC of MTZ could be determined for 882 of the 1293 isolates, and the results for the 10 most abundant genera are shown in Figure 3. MICs for less abundant genera and non-identifiable strains are summarized in Table S1. Resistance of the isolates was assessed based on MTZ resistance breakpoints for anaerobic bacteria defined by CLSI (≥32 µg/mL) [36] and EUCAST (>4 µg/mL) [37]. We measured MIC values for *Gardnerella* across the whole tested range (4–2048 µg/mL) at each visit (Figure 3A). Moreover, 64% (CLSI) and 88% (EUCAST) of *Gardnerella* isolates were resistant already at baseline, prior to MTZ treatment. The percentage of resistant strains increased over the course of this study, from 64% to 80% (CLSI) and from 88% to 94% (EUCAST). The median MIC of *Gardnerella* isolates did not change from baseline to the 6-week visit (64 µg/mL at both visits), but there was a statistically significant increase to 256 µg/mL at the 12-week visit (*p* = 0.0170). PK studies conducted by other groups [31,34] with the same dose of MTZ as used at baseline in CAP083 reported a c_max_ in plasma of ~40 µg/mL. While PK was not measured in CAP083, one may assume that the local MTZ concentration in vaginal fluid reached similar levels as those measured previously in plasma. This would mean that 52% (76/145) of *Gardnerella* strains isolated in this study at baseline had MICs above the estimated c_max_ of MTZ in plasma (and extrapolated for vaginal fluid) (Figure 3A). In another PK study, repeated treatment with 400 mg MTZ for 7 days twice daily led to a steady-state concentration in plasma below 10 µg/mL [35], a concentration only slightly higher than the breakpoint of resistance established by EUCAST for anaerobes. At the 6-week visit, 87% of *Gardnerella* isolates from this study showed an MIC above this reported MTZ plasma steady-state concentration (MIC > 8 µg/mL; note that the treatment in CAP083 was once daily for 5 d and not twice daily for 7 d as in [35]), and the fraction increased to 93% at the 12-week visit. This suggests that MTZ dosing recommended for BV treatment and used in the CAP083 study (single 2 g treatment after baseline, daily 400 mg treatment for 5 d after the 6-week visit) might have been insufficient to remove the vast majority of *Gardnerella* strains (Figure 3A).

All lactobacilli (except one *L. jensenii* isolate), including *L. iners*, were resistant to MTZ at baseline and across all visits (Figure 3B,C). We observed a gradual increase in median MIC values for *L. iners* from 768 µg/mL (baseline) to 1024 µg/mL (6-week visit) and a statistically significant increase to a median of 2048 µg/mL after 12 weeks (*p* = 0.0315). Other genera of the order Lactobacillales were also resistant (100%) (Figure S2). *F. vaginae* isolates were mainly resistant against MTZ with minimal increase throughout this study (78–91% and 89–100% resistance across visits according to CLSI and EUCAST, respectively) (Figure 3D). Of the 23 isolated *Prevotella* strains, a MIC could be determined only for three. At baseline, two susceptible isolates were found, whereas the isolate at the 6-week visit was resistant according to both guidelines. Isolates of the three genera *Finegoldia*, *Peptoniphilus,* and *Anaerococcus*, all three of the class Clostridia, demonstrated greater susceptibility to MTZ, with only 14% (5/37) and 27% (10/37) resistance at the 12-week visit according to CLSI and EUCAST, respectively (Figure 3E). The resistance rate increased by about 10 percentage points from study start to the 12-week visit; however, this difference was not statistically significant (*p* = 0.0931).

### 2.3. MTZ-Resistant Gardnerella Strains Were Isolated from Participants with All Nugent Scores

**Figure 4:**
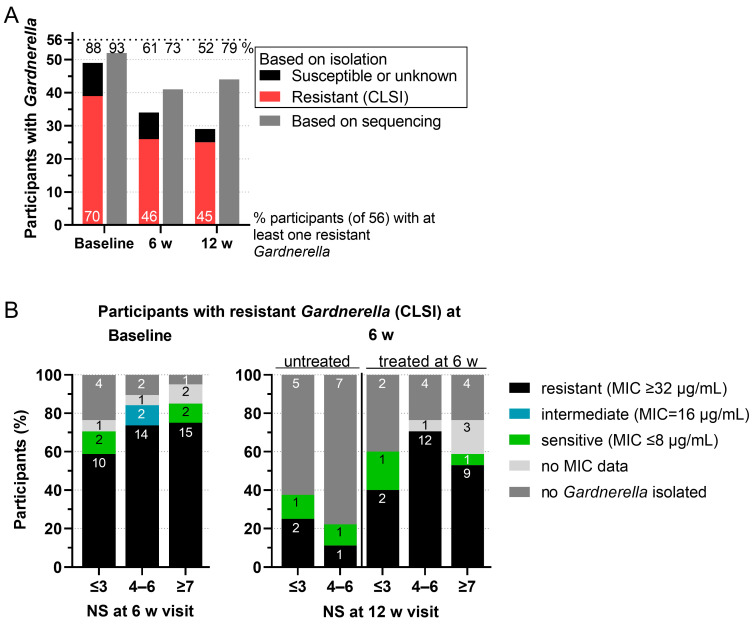
Distribution of MTZ-resistant *Gardnerella* isolates across participants and NS. (**A**) Number of participants with *Gardnerella* based on isolation (red: at least one resistant (CLSI) isolate and black: only susceptible isolates or status unknown) or sequencing (gray, from [13]). The percentage of enrolled participants with *Gardnerella* is stated above the bars. The percentage of participants with at least one resistant isolate is stated within the bars. (**B**) Relation between MTZ-resistant *Gardnerella* isolates and NS of the participants at the following visit. Participants were defined as positive for resistant *Gardnerella* when at least one isolate was tested resistant according to CLSI definitions of breakpoints. The share of participants with at least one resistant *Gardnerella* isolate at the baseline (left) or 6-week visit (right) is plotted over the NS of the respective participants at the following visit. The number of participants is stated within the bars. The same analysis was performed with resistance defined following EUCAST (see Figure S3).

Given the postulated role of *Gardnerella* in BV pathogenesis [19], we investigated whether *Gardnerella* resistance before MTZ treatment was associated with a higher NS of participants at the following visit, i.e., whether MTZ resistance of *Gardnerella* was a predictor of poor treatment outcomes. As shown in Figure 4A, MTZ-resistant *Gardnerella* strains were isolated from 70%, 46%, and 45% of participants at baseline, 6-week visit and 12-week visit, respectively.

As shown in the left panel of Figure 4B, 75% of participants with NS ≥ 7 (BV persistent) at the 6-week visit had had at least one *Gardnerella* strain resistant to MTZ (according to the CLSI definition) at baseline. However, 59% of participants who were BV negative (NS ≤ 3) after 6 weeks (initially cleared) also had at least one resistant *Gardnerella* strain at baseline. A similar analysis was performed for the second MTZ treatment given at the 6-week visit (Figure 4B, right panel). Most participants who were treated again for BV after the 6-week visit had had at least one MTZ-resistant *Gardnerella* strain at the 6-week visit. Specifically, 40%, 71%, and 53% of participants who had NS ≤ 3, NS = 4–6, and NS ≥ 7 at 12 weeks, respectively, were in this category. In contrast, among participants who were BV negative at the 6-week visit, and therefore not retreated, only 25% of those who remained at NS ≤ 3 and 11% of those who shifted to NS = 4–6 at the 12-week visit had had an MTZ-resistant *Gardnerella* strain at the 6-week visit. Similar results but higher overall resistance rates were found when using the EUCAST breakpoint definition in the same analysis (Figure S3).

### 2.4. No Change in Gardnerella Abundance and Participants with Resistant Strains upon MTZ Treatment

**Figure 5:**
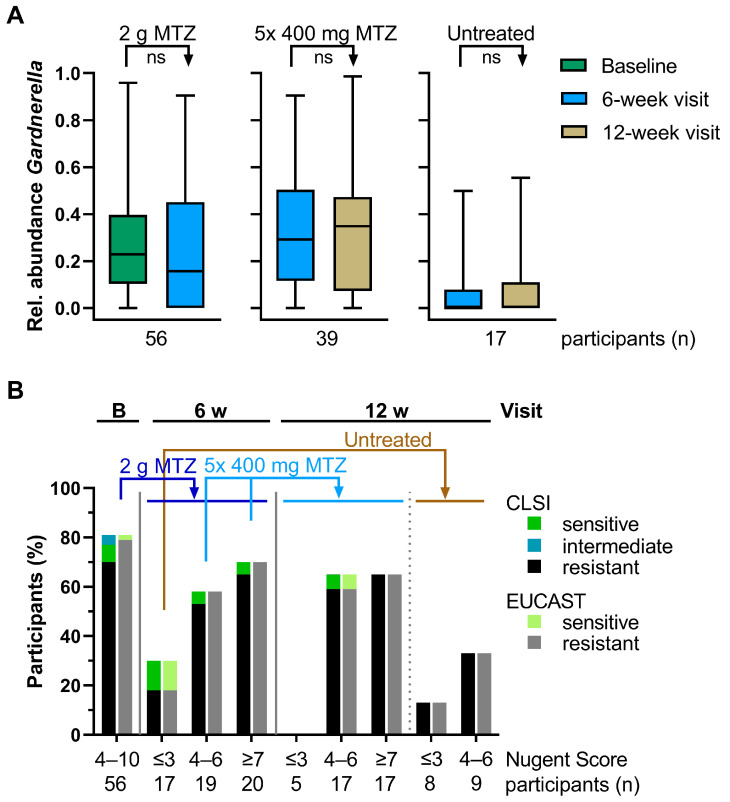
Relative *Gardnerella* abundance and distribution of MTZ resistance pre- and post-treatment. (**A**) Relative abundance of *Gardnerella* determined by 16S rRNA sequencing [13] is shown as box and whiskers (min to max, median as horizontal line) for the indicated groups. After the first visit, all participants received 2 g oral MTZ (left panel). Participants with NS >3 at the 6-week visit received 400 mg MTZ once daily for 5 d (middle panel), whereas the others were not retreated (right panel). Paired t-tests were performed to compare the *Gardnerella* abundance between visits and treatments. All comparisons were not statistically significant (ns). (**B**) Participants were segmented based on their NS at the respective visit and were assigned MTZ-resistant when at least one *Gardnerella* isolate was resistant according to CLSI (MIC ≥ 32 µg/mL) or EUCAST (MIC > 4 µg/mL). Participants were assigned MTZ intermediate (CLSI) when no *Gardnerella* isolate was resistant and at least one had MIC = 16 µg/mL. The y-axis is expressed as a percentage of the total n participants (stated below the graph) in the respective group. Some participants could not be assigned resistant/sensitive because the MIC determination failed or *Gardnerella* was not isolated. MTZ treatment regimens are indicated. B—baseline; ns—not significant; MIC—minimum inhibitory concentration; MTZ—metronidazole; NS—Nugent Score.

Next, we investigated whether the prevalence of *Gardnerella* resistance among participants at baseline influenced the change in its abundance following treatment with MTZ. First, we re-analyzed the 16S rRNA sequencing data on *Gardnerella* abundance previously published by Mtshali and colleagues [13], revealing no significant difference in average relative *Gardnerella* abundance across participants between baseline and the 6-week visit and between the 6-week and 12-week visits (Figure 5A).

We subsequently evaluated whether the prevalence of resistant *Gardnerella* strains changed over time (Figure 5B). At the baseline visit, 70% and 79% of participants had at least one resistant *Gardnerella* strain according to CLSI and EUCAST breakpoints, respectively, with these rates remaining largely unchanged after the two rounds of MTZ treatment. Notably, participants with NS ≤ 3 at 6 weeks showed a lower resistance rate of 18% and sustained a resistance rate below 35% at the 12-week visit. This corresponded with a decreased abundance of *Gardnerella*, as evidenced by the absence of isolated strains in this group at the 12-week follow-up.

### 2.5. Sensitive Gardnerella Strains Were Still Isolated After Treatment with MTZ

**Figure 6:**
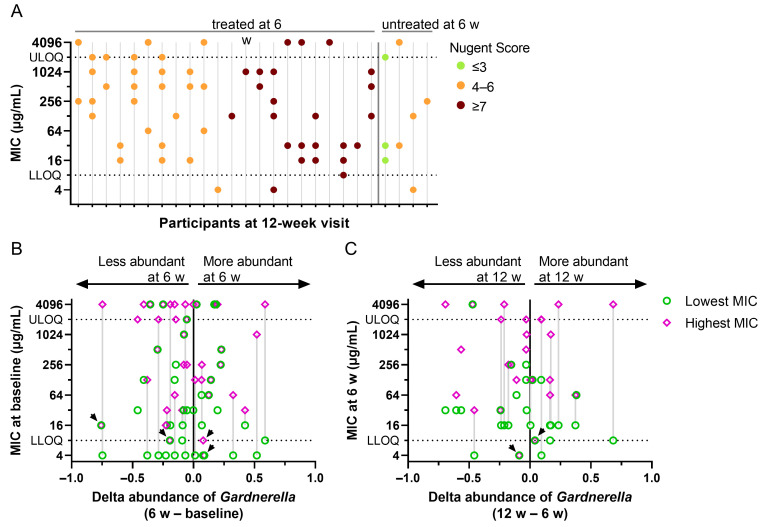
Distribution of MTZ MIC values of *Gardnerella* isolates per participant and difference in relative abundance between later and earlier visits. (**A**) MIC distribution of *Gardnerella* isolates per participant at the 12-week visit treated or untreated at the 6-week visit. Each dot represents the MIC of one isolate. Nugent Score of the participants is color-coded. Participants for whom *Gardnerella* was not isolated or the MIC could not be determined are not shown. (**B**,**C**) The lowest (green) and highest (pink) MIC of MTZ of *Gardnerella* isolates per participant at the baseline (**B**) or 6-week visit (**C**) is plotted over the delta relative abundance (16S rRNA sequencing) of *Gardnerella* between the following and the earlier visit. Lowest and highest MICs are connected with a vertical line when the lowest was below and the highest MIC above the CLSI resistance breakpoint (≥32 µg/mL). Black arrows point to the highest MIC of a participant that is <32 µg/mL. Values below the LLOQ and above the ULOQ were arbitrarily set to 4 µg/mL and 4096 µg/mL, respectively.

We noticed the coexistence of *Gardnerella* strains exhibiting very high and low MICs of MTZ in the same swabs, both pre- and post-treatment (Figure 6A). This was surprising because we expected MTZ to have a stronger bactericidal effect on strains with low MIC than on those with higher MIC, which would result in their selective killing if the strains were independent.

To analyze this further, we plotted the lowest and highest MICs measured for isolates from individual swabs as a function of the difference in relative *Gardnerella* abundance between visits for that participant (Figure 6B,C). If strains were independent, one could expect a reduction in abundance for participants with sensitive *Gardnerella*. However, there was no evident association between the MICs and the change in abundance of *Gardnerella* between visits. Even for swabs where the isolate with the highest measured MIC was still susceptible (below the CLSI resistance breakpoint, labeled by black arrows in Figure 6B,C), the relative *Gardnerella* burden was not systematically reduced after MTZ treatment. Likewise, no systematic change in *Gardnerella* abundance was observed for participants who had isolates with MICs below and above the breakpoint of resistance (labeled with a gray vertical line in Figure 6B,C). These findings suggest that MTZ-resistant and -sensitive *Gardnerella* strains coexist in the vaginal microbiome of individual participants. Furthermore, there may be a mechanism that protects strains with a low MIC from the bactericidal effect of MTZ, at least with the dose regimen applied in CAP083.

### 2.6. MTZ Treatment May Have Increased the MICs of Gardnerella

**Figure 7:**
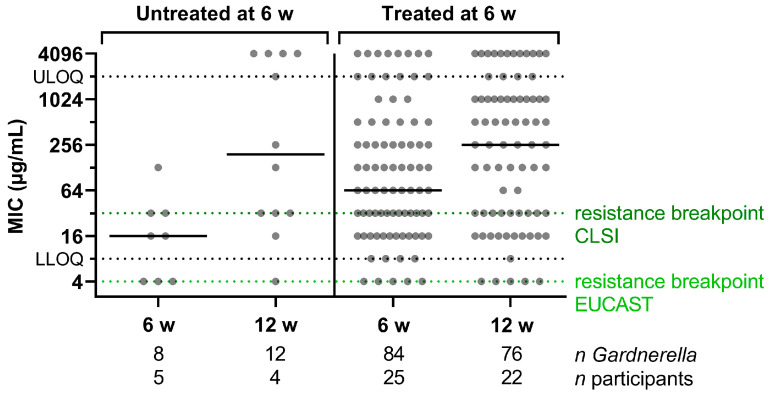
MICs of *Gardnerella* isolates from the 6- and 12-week visits. Participants are grouped depending on their treatment status after the 6-week visit. The number of *Gardnerella* isolates and the corresponding number of participants are stated below the columns. The median is indicated by a horizontal line. MIC—minimum inhibitory concentration.

Given the increase in median MIC of *Gardnerella* isolates from 64 µg/mL to 256 µg/mL between the 6-week and 12-week visits (Figure 3A), we analyzed whether this trend was more pronounced for participants treated with five daily oral doses of 400 mg MTZ after the 6-week visit compared with those not retreated. As shown in Figure 7, the median MIC increased from 64 µg/mL to 256 µg/mL for the isolates from treated participants. However, an increase in median MIC from 16 µg/mL to 192 µg/mL was also observed in non-treated participants between 6 and 12 weeks. Therefore, either the increase in MIC may not be treatment-specific, or the number of participants and isolates analyzed in the non-treated control group was too small to differentiate these effects.

## 3. Discussion

BV is a prevalent condition characterized by dysbiosis of the vaginal microbiome. Despite MTZ being a first-line treatment, many women experience treatment failure and recurrent episodes of BV [13,22,23]. This study utilized culturomics along with previously published 16S rRNA gene sequencing data to assess abundance and resistance levels of multiple BV-associated bacteria before and after MTZ treatment. Our data showed that *Gardnerella* was the most frequently cultured isolate, while *L. iners* was underrepresented compared to its relative abundance in 16S rRNA sequencing.

Notably, 3%, 9%, and 14% of isolates at the baseline, 6-week, and 12-week visits, respectively, were identified as *L. iners*, an organism that is often missed in culture-based studies [38]. This organism has specific nutritional (such as L-cysteine) and environmental requirements that are not adequately replicated in culture media, resulting in its underrepresentation or complete absence in cultured samples [39,40]. Moreover, 16S rRNA sequencing can detect viable but non-culturable states, thus capturing a broad diversity of *L. iners* that may exist in their natural habitats yet remain unobservable through traditional culturing methods. More studies are needed to culture and investigate the role of *L. iners* in the vaginal microbiota as a potential predictive biomarker or risk factor for BV, along with its impact on the mucosal immune microenvironment. The increasing relative abundance of *L. iners* from baseline to follow-up visits is in line with one of the main conclusions of Mtshali and colleagues, that treatment with MTZ strongly increases the overall abundance of this species [13]. Additionally, BVAB1, an uncultured bacterial species [18,41], could not be isolated despite demonstrating a high relative abundance indicated by 16S rRNA gene sequencing [13].

It has been suggested that resistance in *Gardnerella* spp., along with its role as the primary producer of biofilms that hinder antimicrobial penetration, is associated with reduced efficacy of MTZ, contributing to the persistence and recurrence of BV [1,26,42,43,44,45]. Consistent with these findings, this study observed that a large share of *Gardnerella* isolates were resistant to MTZ at baseline, and the resistance level increased throughout this study. Additionally, the stable median MIC from baseline to the 6-week visit, followed by a notable increase among participants receiving multiple doses of MTZ by the 12-week visit, suggests the potential development of resistance as a direct consequence of the treatment. We did not investigate the underlying mechanism of acquired resistance to MTZ, as it was described as complex and multifactorial (reviewed in [46]).

In a recent study, Petrina and colleagues evaluated MTZ susceptibility of vaginal isolates from women in the US [30]. They reported a median MIC of MTZ of 8 µg/mL for 110 *Gardnerella* isolates, with 27% of strains being classified as MTZ-resistant according to CLSI. This contrasts with our study, in which we found a median MIC of 64 µg/mL and 64% resistant *Gardnerella* isolates at the baseline. The differences between the two studies may come from the geographic locations but also from the study populations, which in the case of Petrina et al. also included women without BV.

The majority of isolated strains at baseline (52%, 76/145) exhibited MICs higher than the maximum plasma concentration of MTZ reported to be reached after oral dosing of 2 g MTZ (~40 µg/mL) in PK studies conducted by other groups [31,34]. At the 6-week visit, 91% (84/92) of determined MIC values exceeded the reported steady-state plasma concentration after dosing twice daily with 400 mg for 7 days (~7 µg/mL) [35]). The plasma concentrations reached in CAP083 participants may have been even lower, because the participants were only dosed once a day for 5 days. The reported levels of MTZ in plasma after similar dose regimens as in CAP083, along with the high median MICs for MTZ among bacterial isolates, may explain the low change in the relative abundance of *Gardnerella* observed in CAP083 after treatment. This is also reflected in the low cure rate of only 23% at the 12-week follow-up. Instead, the 5-day treatment after the 6-week visit significantly increased the median MIC of *Gardnerella* and *L. iners* isolates. Consistently, previous studies have reported recurrence rates of BV of up to 60% within 12 months after oral MTZ treatment, with MTZ resistance being a contributing factor [2,8,9,28]. Our analysis indicates a certain association between pre-existing *Gardnerella* resistance and poor outcomes of MTZ treatment. However, the high share of participants with at least one MTZ-resistant *Gardnerella* strain, which was 70% and 46% before the first and second treatment, respectively, may have confounded a clear outcome.

Furthermore, we observed a relatively stable average abundance of *Gardnerella* in participants throughout this study (re-analyzed data from [13]) across NS states. The low reduction in *Gardnerella* and the low cure rate of 23% in CAP083 is consistent with (but obviously does not prove) a keystone role of *Gardnerella* in BV [19]. Other studies report 4-week cure rates of above 60% after oral MTZ therapy [47], but high recurrence rates within 12 months [13].

A spread of MIC values from sensitive to highly resistant *Gardnerella* strains was observed within individual swabs. This spread was also found at the 12-week visit, indicating that i) strains with different MTZ sensitivity coexist in the vaginal microbiome and that ii) sensitive strains can persist after treatment when resistant strains are present. This might indicate that MTZ-resistant strains protect sensitive ones, at least at the dose regimen applied in CAP083. A mechanistic model that would explain these observations could be that the outer layers of the biofilm reduce the MTZ exposure of the inner layers, meaning that resistant bacteria might be most abundant in the outer layers of the biofilm. Recent studies reported that all currently known *Gardnerella* species can coexist in single microbiomes [48], that *Gardnerella* species diversity is high in BV [49], and that different *Gardnerella* species may contribute differently to BV [50], adding another layer of complexity. Consistently, the re-analysis of CAP083 NS and 16S rRNA sequencing data [13] indicated a lack of significant changes in the average *Gardnerella* abundance across all participants before and after treatment.

This study encountered several limitations that may influence the interpretation of its findings. Firstly, the reliance on existing 16S rRNA sequencing data may restrict our ability to fully capture the complexity of the vaginal microbiome, particularly for underrepresented species like *L. iners*. Furthermore, the isolation of only eight isolates per participant and timepoint may not adequately reflect the full diversity of strains present in the vaginal microbiome. Additionally, we were unable to differentiate between *Gardnerella* species, which may have distinct roles in BV and could contribute variably to treatment outcomes. Lastly, the study’s participants may have had a history of BV treatment prior to enrollment, which could skew results by leading to higher rates of *Gardnerella* detection. This prior treatment history may influence the observed resistance patterns within the cohort, thereby affecting the overall conclusions regarding antibiotic resistance and treatment efficacy.

To better understand the pathogenesis of BV, future studies are needed to investigate the coexistence of sensitive and resistant bacteria. Also, knowledge of the most important bacterial genera that need to be targeted to allow the vaginal microbiome to shift away from CSTs III and IV needs to be deepened to develop an improved therapy. Our study highlights the need for more effective treatment options due to widespread resistance to the current standard of care in bacteria associated with disease relapse and recurrence.

## 4. Materials and Methods

### 4.1. Sample Collection

Dry vaginal swabs collected from 56 South African women diagnosed with BV (NS ≥ 4) and any STIs other than HIV, as part of the CAP083 study, were used for 16S rRNA sequencing [13] and later to isolate bacterial strains. In the CAPRISA 083 cohort, BV diagnosis and treatment were based on clinical symptoms and NS, in accordance with national guidelines prioritizing the treatment of symptomatic BV. All enrolled participants in this study were Black African women. Following baseline sampling, participants received a single oral dose of 2 g MTZ for the treatment of BV, and additional treatment for STIs was provided if indicated. After 6 weeks, participants with NS ≥ 4 (70%, 39/56) received a subsequent 5-day treatment of MTZ (400 mg orally) following a second swab collection. A final swab was taken 12 weeks after the post-treatment. CSTs were assigned using the VALENCIA classification [51]. More detailed information on the characteristics of the study population and the changes in CSTs following MTZ treatment can be found in the initial description of the CAP083 study [13].

### 4.2. Isolation and Identification of Bacterial Strains from Vaginal Swabs

Dry vaginal swabs (156) from CAP083 [13], collected at baseline and 6 and 12 weeks post-MTZ treatment (*n* = 56 at each visit) and stored at −80 °C, were reconstituted in culture media designed to promote the growth of known BV-associated bacteria, as well as lactobacilli. Swabs were reconstituted in 1 mL of pH-unadjusted 20% New York City broth (NYCB) supplemented with 2% horse serum (Thermo Scientific, Vienna, Austria) in 0.9% saline. The reconstituted samples were plated on blood agar (GSA, BD, Temse, Belgium) and eight randomly picked colonies were restreaked to obtain single colonies. Identification of isolated bacteria was conducted using matrix-assisted laser desorption time-of-flight mass spectrometry (MALDI-TOF MS, Bruker Corporation, MA, USA) at the University of Natural Resources and Life Sciences (BOKU) in Vienna, Austria. Cultivation was carried out in tightly sealed plastic boxes using an anaerobic atmosphere generation bag (Fisher Scientific, Schwerte, Germany) and an anaerobic indicator at 37 °C for 72 h. For susceptibility testing, a 10 µL loop (VWR, Vienna, Austria) of bacteria was resuspended in 300 µL of unadjusted NYCB in a 2 mL deep well plate (VWR, Vienna, Austria) and diluted 1:10 in medium. Prior to storage at −80 °C, the bacterial suspension was supplemented with glycerol to achieve a final concentration of 25%, ensuring viability upon thawing.

### 4.3. Susceptibility Testing of Bacterial Isolates to MTZ

Susceptibility of bacterial isolates to MTZ was determined using a high-throughput broth microdilution method in monoplicates. In a 96-well flat-bottom plate (Brand, Wertheim, Germany), 50 µL of the bacterial suspension was mixed with 50 µL of a two-fold dilution series of MTZ (Paulus Pharmacy, Vienna, Austria) in NYCB, resulting in final concentrations ranging from 4 to 2048 µg/mL. The preparation of the dilution series and the pipetting of the reaction plates were automated using a Freedom EVO150 robot (Tecan, Männedorf, Switzerland). The bacterial input was not adjusted; however, it was estimated to be approximately 10^6^ CFU/mL based on preliminary tests. Optical density (OD) measurements were taken at 610 nm using a plate reader (Tecan, Männedorf, Switzerland), and the reaction plates were statically incubated in anaerobic conditions at 37 °C for 48 h. After incubation, OD was measured again and blanked against the respective time zero measurement. The MIC of MTZ was defined as the lowest concentration that inhibited bacterial growth. MIC determinations were performed only for isolates with a growth control (GC, no MTZ) achieving an OD ≥ 0.06. In case GC ≥ 0.2 OD, MIC was determined as the lowest concentration at which OD < 0.05. In case GC < 0.2, MIC was determined as the lowest concentration at which OD < 30% of GC. Resistance of the isolates was assessed based on MTZ breakpoints for anaerobes defined by CLSI (Clinical and Laboratory Standards Institute, ≥32 µg/mL) [36] and EUCAST (European Committee on Antimicrobial Susceptibility Testing, >4 µg/mL) [37].

### 4.4. Statistical Analyses

All statistical calculations were conducted using GraphPad Prism 9.5.1. Ordinary one-way ANOVA was performed on log2-transformed MIC values, followed by Šidák’s multiple comparison test to compare the MICs between the baseline and the 6-week visit, as well as between the 6-week visit and the 12-week visit. Paired *t*-tests were performed to compare the *Gardnerella* abundance between indicated groups. *p*-values > 0.05 were considered not significant.

## 5. Conclusions

MTZ is a first-line treatment for BV, but many women experience treatment failure and recurrent episodes of BV. Analysis of susceptibility of isolated bacterial strains from vaginal swabs of women diagnosed with BV showed a high rate of MTZ-resistant strains prior to and after MTZ-treatment. An increase of the median MIC values of *Gardnerella* over the course of the study suggests the development of resistance as a consequence of the treatment with a potentially insufficient MTZ dose regimen. To gain a better understanding of recurrent BV, future studies are needed to investigate the coexistence of sensitive and resistant strains, and to identify the most important bacterial genera to be targeted to shift an imbalanced vaginal microbiome into a stable state.

## Data Availability

Data are contained within the article and Supplementary Materials.

## Supplementary Materials

The following supporting information can be downloaded at: https://www.mdpi.com/article/10.3390/antibiotics13121217/s1. Figure S1: Bacterial strains isolated from participants assigned to the indicated community state types. The genus *Lactobacillus* is divided into *Lactobacillus* excluding *L. iners* (dark green) and *L. iners* only (light green). Genera belonging to the class Clostridia are shown in dark gray, and genera of the order Lactobacillales are shown in light gray. Isolates that could not be identified are labeled as “not identifiable”. The number of participants in each community state type (CST) is stated above the bars. Figure S2: Minimum inhibitory concentration of metronidazole on Lactobacillales. Included are Lactobacillales other than the genus *Lactobacillus* (shown in Figure 3B,C). The number of tested isolates (n) is stated above the graph. Values above the ULOQ were arbitrarily set to 4096 µg/mL. Figure S3: Relation between MTZ-resistant *Gardnerella* and Nugent Score of participants at the following visit. Participants were assigned as resistant when at least one *Gardnerella* isolate was tested resistant according to the EUCAST definition of breakpoints. The resistance state of *Gardnerella* isolates at the baseline (left) or 6-week visit (right) is plotted over the NS of respective participants at the following visit. The number of participants is stated within the bars. Table S1: MICs of MTZ of not identifiable isolates and genera not shown in Figure 3 and Figure S2.

## References

[1] Bradshaw C.S., Sobel J.D. (2016). Current Treatment of Bacterial Vaginosis-Limitations and Need for Innovation. J. Infect. Dis..

[2] Muzny C.A., Sobel J.D. (2022). The Role of Antimicrobial Resistance in Refractory and Recurrent Bacterial Vaginosis and Current Recommendations for Treatment. Antibiotics.

[3] Torrone E.A., Morrison C.S., Chen P.-L., Kwok C., Francis S.C., Hayes R.J., Looker K.J., McCormack S., McGrath N., van de Wijgert J.H.H.M. (2018). Prevalence of sexually transmitted infections and bacterial vaginosis among women in sub-Saharan Africa: An individual participant data meta-analysis of 18 HIV prevention studies. PLoS Med..

[4] Shipitsyna E., Roos A., Datcu R., Hallen A., Fredlund H., Jensen J.S., Engstrand L., Unemo M. (2013). Composition of the vaginal microbiota in women of reproductive age—Sensitive and specific molecular diagnosis of bacterial vaginosis is possible?. PLoS ONE.

[5] Swidsinski S., Moll W.M., Swidsinski A. (2023). Bacterial Vaginosis-Vaginal Polymicrobial Biofilms and Dysbiosis. Dtsch. Arztebl. Int..

[6] Unemo M., Bradshaw C.S., Hocking J.S., de Vries H.J.C., Francis S.C., Mabey D., Marrazzo J.M., Sonder G.J.B., Schwebke J.R., Hoornenborg E. (2017). Sexually transmitted infections: Challenges ahead. Lancet Infect. Dis..

[7] Sobel J.D., Ferris D., Schwebke J., Nyirjesy P., Wiesenfeld H.C., Peipert J., Soper D., Ohmit S.E., Hillier S.L. (2006). Suppressive antibacterial therapy with 0.75% metronidazole vaginal gel to prevent recurrent bacterial vaginosis. Am. J. Obstet. Gynecol..

[8] Bradshaw C.S., Morton A.N., Hocking J., Garland S.M., Morris M.B., Moss L.M., Horvath L.B., Kuzevska I., Fairley C.K. (2006). High recurrence rates of bacterial vaginosis over the course of 12 months after oral metronidazole therapy and factors associated with recurrence. J. Infect. Dis..

[9] Muzny C.A., Sobel J.D. (2023). Understanding and Preventing Recurring Bacterial Vaginosis: Important Considerations for Clinicians. Int. J. Womens Health.

[10] Ravel J., Gajer P., Abdo Z., Schneider G.M., Koenig S.S., McCulle S.L., Karlebach S., Gorle R., Russell J., Tacket C.O. (2011). Vaginal microbiome of reproductive-age women. Proc. Natl. Acad. Sci. USA.

[11] Lebeer S., Ahannach S., Gehrmann T., Wittouck S., Eilers T., Oerlemans E., Condori S., Dillen J., Spacova I., Vander Donck L. (2023). A citizen-science-enabled catalogue of the vaginal microbiome and associated factors. Nat. Microbiol..

[12] Hugerth L.W., Krog M.C., Vomstein K., Du J., Bashir Z., Kaldhusdal V., Fransson E., Engstrand L., Nielsen H.S., Schuppe-Koistinen I. (2024). Defining Vaginal Community Dynamics: Daily microbiome transitions, the role of menstruation, bacteriophages, and bacterial genes. Microbiome.

[13] Mtshali A., San J.E., Osman F., Garrett N., Balle C., Giandhari J., Onywera H., Mngomezulu K., Mzobe G., de Oliveira T. (2021). Temporal Changes in Vaginal Microbiota and Genital Tract Cytokines Among South African Women Treated for Bacterial Vaginosis. Front. Immunol..

[14] Zheng N., Guo R., Wang J., Zhou W., Ling Z. (2021). Contribution of *Lactobacillus iners* to Vaginal Health and Diseases: A Systematic Review. Front. Cell. Infect. Microbiol..

[15] Petrova M.I., Reid G., Vaneechoutte M., Lebeer S. (2017). Lactobacillus iners: Friend or Foe?. Trends Microbiol..

[16] Fettweis J.M., Brooks J.P., Serrano M.G., Sheth N.U., Girerd P.H., Edwards D.J., Strauss J.F., Jefferson K.K., Buck G.A., The Vaginal Microbiome Consortium (2014). Differences in vaginal microbiome in African American women versus women of European ancestry. Microbiology.

[17] Zhou X., Brown C.J., Abdo Z., Davis C.C., Hansmann M.A., Joyce P., Foster J.A., Forney L.J. (2007). Differences in the composition of vaginal microbial communities found in healthy Caucasian and black women. ISME J..

[18] Holm J.B., France M.T., Ma B., McComb E., Robinson C.K., Mehta A., Tallon L.J., Brotman R.M., Ravel J. (2020). Comparative Metagenome-Assembled Genome Analysis of “*Candidatus* Lachnocurva vaginae”, Formerly Known as Bacterial Vaginosis-Associated Bacterium−1 (BVAB1). Front. Cell. Infect. Microbiol..

[19] Muzny C.A., Taylor C.M., Swords W.E., Tamhane A., Chattopadhyay D., Cerca N., Schwebke J.R. (2019). An Updated Conceptual Model on the Pathogenesis of Bacterial Vaginosis. J. Infect. Dis..

[20] Machado A., Cerca N. (2015). Influence of Biofilm Formation by *Gardnerella vaginalis* and Other Anaerobes on Bacterial Vaginosis. J. Infect. Dis..

[21] Workowski K.A., Bachmann L.H., Chan P.A., Johnston C.M., Muzny C.A., Park I., Reno H., Zenilman J.M., Bolan G.A. (2021). Sexually Transmitted Infections Treatment Guidelines, 2021. MMWR Recomm. Rep..

[22] Joag V., Obila O., Gajer P., Scott M.C., Dizzell S., Humphrys M., Shahabi K., Huibner S., Shannon B., Tharao W. (2019). Impact of Standard Bacterial Vaginosis Treatment on the Genital Microbiota, Immune Milieu, and Ex Vivo Human Immunodeficiency Virus Susceptibility. Clin. Infect. Dis..

[23] Verwijs M.C., Agaba S.K., Darby A.C., van de Wijgert J. (2020). Impact of Oral Metronidazole Treatment on the Vaginal Microbiota and Correlates of Treatment Failure. Am. J. Obstet. Gynecol..

[24] Bostwick D.G., Woody J., Hunt C., Budd W. (2016). Antimicrobial resistance genes and modelling of treatment failure in bacterial vaginosis: Clinical study of 289 symptomatic women. J. Med. Microbiol..

[25] Barrons R., Tassone D. (2008). Use of *Lactobacillus* probiotics for bacterial genitourinary infections in women: A review. Clin. Ther..

[26] Swidsinski A., Mendling W., Loening-Baucke V., Swidsinski S., Dörffel Y., Scholze J., Lochs H., Verstraelen H. (2008). An adherent *Gardnerella vaginalis* biofilm persists on the vaginal epithelium after standard therapy with oral metronidazole. Am. J. Obstet. Gynecol..

[27] Abbe C., Mitchell C.M. (2023). Bacterial vaginosis: A review of approaches to treatment and prevention. Front. Reprod. Health.

[28] Landlinger C., Oberbauer V., Podpera Tisakova L., Schwebs T., Berdaguer R., Van Simaey L., Vaneechoutte M., Corsini L. (2022). Preclinical Data on the *Gardnerella*-Specific Endolysin PM-477 Indicate Its Potential to Improve the Treatment of Bacterial Vaginosis through Enhanced Biofilm Removal and Avoidance of Resistance. Antimicrob. Agents Chemother..

[29] Landlinger C., Tisakova L., Oberbauer V., Schwebs T., Muhammad A., Latka A., Van Simaey L., Vaneechoutte M., Guschin A., Resch G. (2021). Engineered Phage Endolysin Eliminates *Gardnerella* Biofilm without Damaging Beneficial Bacteria in Bacterial Vaginosis Ex Vivo. Pathogens.

[30] Petrina M.A.B., Cosentino L.A., Rabe L.K., Hillier S.L. (2017). Susceptibility of bacterial vaginosis (BV)-associated bacteria to secnidazole compared to metronidazole, tinidazole and clindamycin. Anaerobe.

[31] Loft S., Dossing M., Poulsen H.E., Sonne J., Olesen K.L., Simonsen K., Andreasen P.B. (1986). Influence of dose and route of administration on disposition of metronidazole and its major metabolites. Eur. J. Clin. Pharmacol..

[32] National Department of Health, Republic of South Africa Management Guidelines, Sexually Transmitted Infections. https://www.health.gov.za/wp-content/uploads/2020/11/sti-guidelines-27-08-19.pdf.

[33] Garrett N.J., Osman F., Maharaj B., Naicker N., Gibbs A., Norman E., Samsunder N., Ngobese H., Mitchev N., Singh R. (2018). Beyond syndromic management: Opportunities for diagnosis-based treatment of sexually transmitted infections in low- and middle-income countries. PLoS ONE.

[34] Amon I., Amon K., Hüller H. (1978). Pharmacokinetics and therapeutic efficacy of metronidazole at different dosages. Int. J. Clin. Pharmacol. Biopharm..

[35] Jensen J.C., Gugler R. (1983). Single- and multiple-dose metronidazole kinetics. Clin. Pharmacol. Ther..

[36] Clinical and Laboratory Standards Institute (2024). Performance Standards for Antimicrobial Susceptibility Testing.

[37] The European Committee on Antimicrobial Susceptibility Testing (2024). Breakpoint Tables for Interpretation of MICs and Zone Diamters. http://www.eucast.org.

[38] Vaneechoutte M. (2017). *Lactobacillus iners*, the unusual suspect. Res. Microbiol..

[39] De Man J.C., Rogosa M., Sharpe M.E. (1960). A medium for the cultivation of lactobacilli. J. Appl. Bacteriol..

[40] Bloom S.M., Mafunda N.A., Woolston B.M., Hayward M.R., Frempong J.F., Abai A.B., Xu J., Mitchell A.J., Westergaard X., Hussain F.A. (2022). Cysteine dependence of *Lactobacillus iners* is a potential therapeutic target for vaginal microbiota modulation. Nat. Microbiol..

[41] Srinivasan S., Munch M.M., Sizova M.V., Fiedler T.L., Kohler C.M., Hoffman N.G., Liu C., Agnew K.J., Marrazzo J.M., Epstein S.S. (2016). More Easily Cultivated Than Identified: Classical Isolation With Molecular Identification of Vaginal Bacteria. J. Infect. Dis..

[42] Nagaraja P. (2008). Antibiotic resistance of *Gardnerella vaginalis* in recurrent bacterial vaginosis. Indian J. Med. Microbiol..

[43] Li T., Zhang Z., Wang F., He Y., Zong X., Bai H., Liu Z. (2020). Antimicrobial Susceptibility Testing of Metronidazole and Clindamycin against *Gardnerella vaginalis* in Planktonic and Biofilm Formation. Can. J. Infect. Dis. Med. Microbiol..

[44] Bannatyne R.M., Smith A.M. (1998). Recurrent bacterial vaginosis and metronidazole resistance in *Gardnerella vaginalis*. Sex. Transm. Infect..

[45] Schuyler J.A., Mordechai E., Adelson M.E., Sobel J.D., Gygax S.E., Hilbert D.W. (2016). Identification of intrinsically metronidazole-resistant clades of *Gardnerella vaginalis*. Diagn. Microbiol. Infect. Dis..

[46] Alauzet C., Lozniewski A., Marchandin H. (2019). Metronidazole resistance and nim genes in anaerobes: A review. Anaerobe.

[47] Koumans E.H., Markowitz L.E., Hogan V., Group C.B.W. (2002). Indications for therapy and treatment recommendations for bacterial vaginosis in nonpregnant and pregnant women: A synthesis of data. Clin. Infect. Dis..

[48] Berman H.L., Goltsman D.S.A., Anderson M., Relman D.A., Callahan B.J. (2024). *Gardnerella* diversity and ecology in pregnancy and preterm birth. mSystems.

[49] Munch M.M., Strenk S.M., Srinivasan S., Fiedler T.L., Proll S., Fredricks D.N. (2024). *Gardnerella* Species and Their Association With Bacterial Vaginosis. J. Infect. Dis..

[50] Hill J.E., Albert A.Y.K., Group V.R. (2019). Resolution and Cooccurrence Patterns of *Gardnerella leopoldii*, *G. swidsinskii*, *G. piotii*, and *G. vaginalis* within the Vaginal Microbiome. Infect. Immun..

[51] France M.T., Ma B., Gajer P., Brown S., Humphrys M.S., Holm J.B., Waetjen L.E., Brotman R.M., Ravel J. (2020). VALENCIA: A nearest centroid classification method for vaginal microbial communities based on composition. Microbiome.

