# Supplementary material for: Evaluation of Metronidazole Resistance of Vaginal Swab Isolates from South African Women Treated for Bacterial Vaginosis
Source: Antibiotics (Basel). 2024 Dec 14;13(12):1217. doi: 10.3390/antibiotics13121217 (PMC11672489; doi:10.3390/antibiotics13121217)
Supplement: Supplementary file 1 [file antibiotics-13-01217-s001.zip › antibiotics-3341477-supplementary.pdf]

Supplementary Data

Figure S1

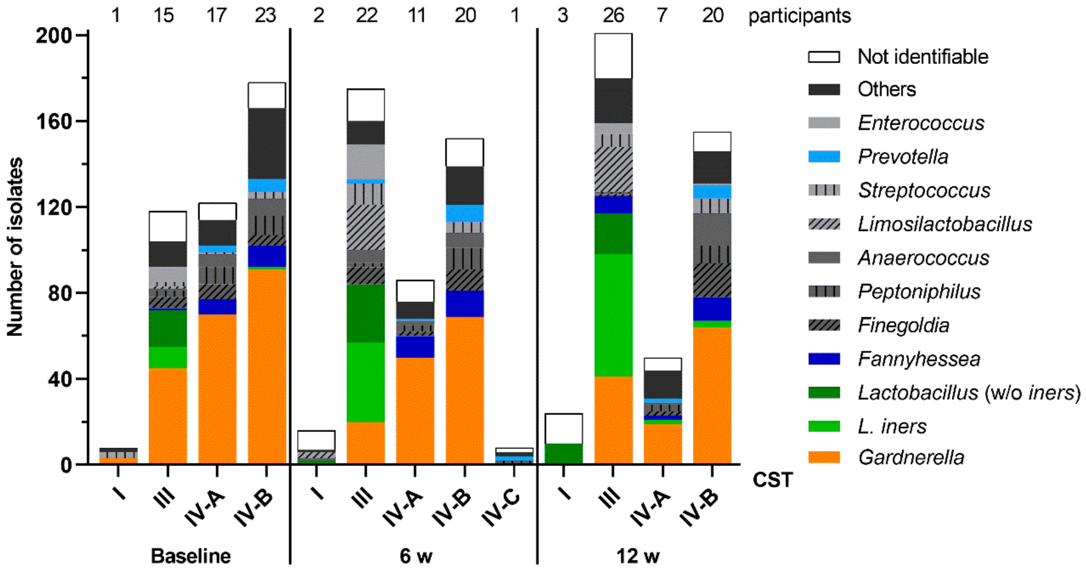

Figure S1. Bacterial strains isolated from participants assigned to the indicated community state types. The genus *Lactobacillus* is divided into *Lactobacillus* excluding *L. iners* (dark green) and *L. iners* only (light green). Genera belonging to the class Clostridia are shown in dark gray, and genera of the order Lactobacillales are shown in light gray. Isolates that could not be identified are labeled as "not identifiable". The number of participants in each community state type (CST) is stated above the bars.

Figure S2

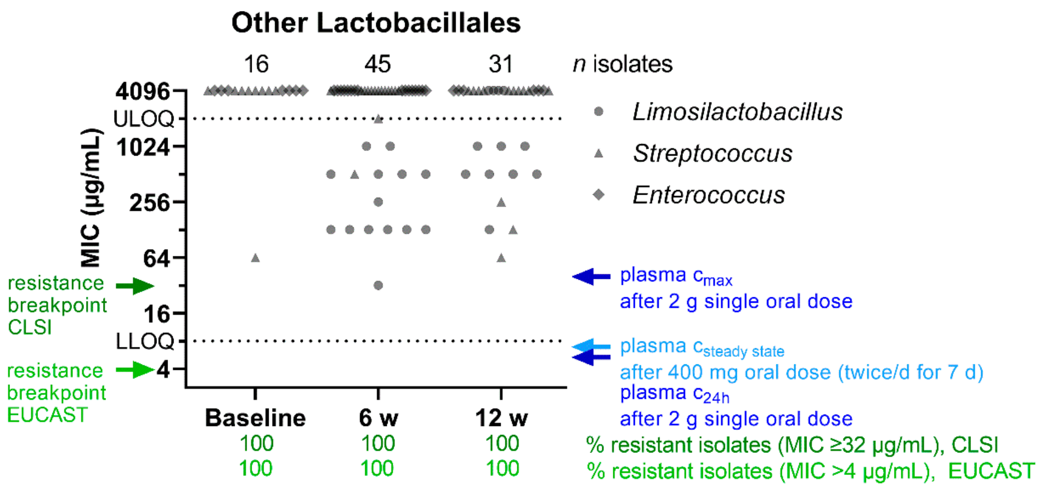

Figure S2. Minimum inhibitory concentration of metronidazole on Lactobacillales. Included are Lactobacillales other than the genus *Lactobacillus* (shown in Figure 3 B,C). The number of tested isolates (n) is stated above the graph. Values above the ULOQ were arbitrarily set to 4096 µg/mL. LLOQ—lower limit of quantification; MIC—minimum inhibitory concentration; ULOQ—upper limit of quantification.

Figure S3

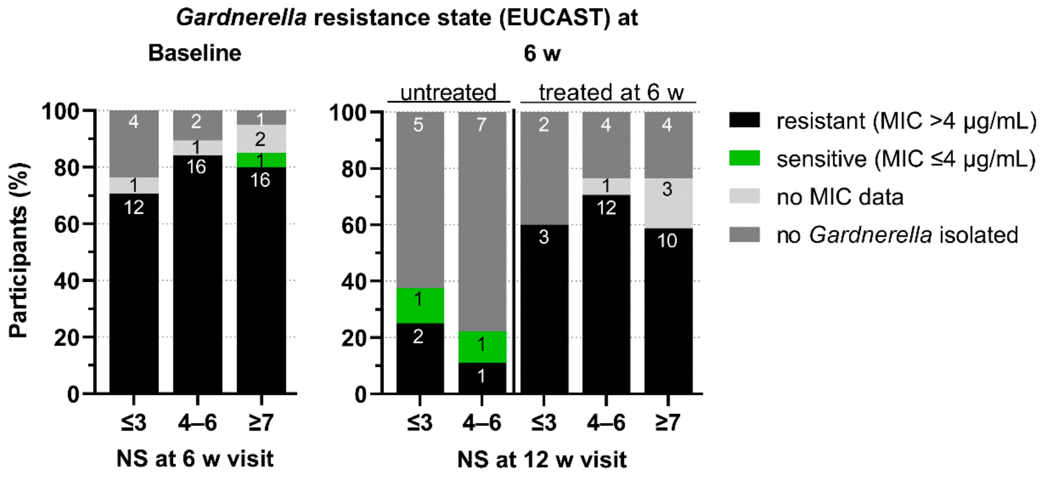

Figure S3. Relation between MTZ-resistant *Gardnerella* and Nugent Score of participants at the following visit. Participants were assigned resistant when at least one *Gardnerella* isolate was tested resistant according to the EUCAST definition of breakpoints. The resistance state of *Gardnerella* isolates at the baseline (left) or 6-week visit (right) is plotted over the NS of respective participants at the following visit. The number of participants is stated within the bars.

Table S1. MICs of MTZ of not identifiable isolates and genera not shown in Figures 3 and S2.

		MIC (µg/mL) at visit		
**Genus**	**Isolate ID**	**Baseline**	**6 week**	**12 week**
Actinomyces	120093_1020_7		4096	
	120093_1030_2			4096
	120093_1030_8			4096
Aerococcus	120089_1000_1	256		
	120089_1000_4	4096		
	120093_1030_1			512
	120096_1000_2	4096		
	120096_1000_3	1024		
	120098_1030_5			4
Bacteroides	120003_1020_7		64	
Bifidobacterium	120019_1000_3	4096		
	120020_1000_1	4096		
	120023_1000_1	16		
	120023_1000_5	8		
	120098_1020_2		16	
Corynebacterium	120019_1020_7		4096	
	120022_1020_4		4096	
	120022_1020_7		128	
	120043_1020_8		4096	
	120096_1000_4	4096		
	120096_1020_1		4096	
	120109_1020_6		4096	
	120125_1030_3			4096
	120142_1000_3	4096		
	120187_1030_6			4096
	120187_1030_8			4096
Criibacterium	120057_1030_4			4
Cutibacterium	120013_1000_7	4096		
	120030_1030_5			4096
	120037_1030_3			4096
	120044_1030_3			4096
	120074_1000_1	4096		
	120074_1000_2	4096		
	120074_1000_4	4096		
	120074_1000_5	4096		
	120074_1000_6	4096		
Dermabacter	120102_1000_4	4096		
Facklamia	120017_1020_8		4096	
	120047_1030_1			512
	120047_1030_2			1024

		MIC (μg/mL) at visit		
**Genus**	**Isolate ID**	**Baseline**	**6 week**	**12 week**
	120081_1020_5		256	
	120125_1030_2			16
Levyella	120044_1000_7	4		
	120057_1030_3			4096
	120057_1030_5			4096
	120057_1030_6			4096
	120057_1030_7			4
Ligilactobacillus	120013_1020_5		2048	
	120013_1020_6		2048	
Metamycoplasma	120078_1030_6			4096
Peptostreptococcus	120044_1000_3	4		
	120057_1030_1			4
	120083_1030_4			4
Propionibacterium	120030_1030_1			4096
	120030_1030_6			4096
Schaalia	120074_1020_6		4096	
	120074_1020_7		4096	
	120078_1020_4		512	
	120093_1030_5			4096
	120204_1000_8	4096		
Staphylococcus	120019_1000_1	4096		
	120019_1000_8	4096		
	120024_1000_5	2048		
	120030_1020_3		4096	
	120030_1030_3			1024
	120030_1030_7			2048
	120030_1030_8			1024
	120037_1030_2			2048
	120067_1000_1	2048		
	120081_1000_1	256		
	120089_1020_1		4096	
	120089_1020_4		4096	
	120089_1020_5		4096	
	120125_1030_1			1024
	120125_1030_8			2048
	120144_1000_8	2048		
	120187_1000_5	1024		
	120187_1000_8	2048		
Winkia	120003_1030_3			2048
	120003_1030_7			2048
	120013_1000_3	128		
	120013_1000_5	256		

		MIC (µg/mL) at visit		
**Genus**	**Isolate ID**	**Baseline**	**6 week**	**12 week**
	120013_1020_2		256	
	120013_1020_4		1024	
	120013_1030_4			128
	120013_1030_7			128
	120024_1000_8	4096		
	120037_1000_8	256		
	120045_1030_7			1024
	120049_1000_2	256		
	120096_1000_1	4096		
	120096_1000_6	4096		
	120109_1000_4	512		
Non-identifiable	120003_1030_1			4096
	120013_1000_6	256		
	120013_1030_1			128
	120015_1020_1		64	
	120015_1020_2		16	
	120015_1020_4		128	
	120019_1020_2		4096	
	120019_1020_3		2048	
	120019_1020_5		4096	
	120019_1020_8		4096	
	120019_1030_7			4096
	120023_1000_7	4096		
	120024_1020_2		4096	
	120036_1000_4	32		
	120037_1020_8		256	
	120043_1030_2			4096
	120044_1020_4		128	
	120044_1030_6			4096
	120045_1030_5			4096
	120049_1000_6	1024		
	120049_1020_5		4096	
	120049_1020_8		4096	
	120052_1020_3		256	
	120052_1020_4		4	
	120057_1020_6		32	
	120058_1020_2		4096	
	120058_1020_6		4096	
	120058_1030_2			4096
	120058_1030_3			4096
	120058_1030_4			4096
	120058_1030_5			4096

		MIC (μg/mL) at visit		
**Genus**	**Isolate ID**	**Baseline**	**6 week**	**12 week**
	120058_1030_6			4096
	120058_1030_7			4096
	120062_1000_8	4096		
	120062_1030_2			4096
	120067_1000_2	4096		
	120067_1000_4	4096		
	120067_1000_7	4096		
	120067_1020_1		4096	
	120067_1020_3		4096	
	120067_1020_5		4096	
	120067_1020_8		4096	
	120067_1030_1			4096
	120067_1030_2			4096
	120067_1030_3			4096
	120067_1030_4			4096
	120067_1030_5			4096
	120067_1030_6			4096
	120067_1030_8			4096
	120070_1000_3	2048		
	120070_1000_7	16		
	120079_1000_3	16		
	120079_1030_4			512
	120081_1000_8	4096		
	120089_1030_1			64
	120089_1030_4			64
	120090_1020_1		256	
	120090_1020_7		128	
	120093_1000_1	4096		
	120099_1020_4		128	
	120099_1020_5		32	
	120099_1020_6		16	
	120099_1020_7		32	
	120099_1030_3			32
	120102_1020_4		4096	
	120109_1000_6	2048		
	120111_1000_2	4		
	120111_1000_3	4		
	120111_1000_5	4		
	120111_1000_6	4		
	120111_1000_7	4		
	120111_1020_4		64	
	120111_1020_8		128	

Genus	Isolate ID	MIC (μg/mL) at visit		
		Baseline	6 week	12 week
	120184_1000_8	512		
	120204_1030_1			512
